# Copper Availability Influences the Transcriptomic Response of *Candida albicans* to Fluconazole Stress

**DOI:** 10.1093/g3journal/jkab065

**Published:** 2021-03-08

**Authors:** Elizabeth W Hunsaker, Chen-Hsin Albert Yu, Katherine J Franz

**Affiliations:** 1 Department of Chemistry, French Family Science Center, Duke University, Durham, NC 27708, USA; 2 Institute of Molecular Biology, Academia Sinica, Taipei, Taiwan

**Keywords:** copper, antifungal, RNA-seq, metals in medicine, metal homeostasis

## Abstract

The ability of pathogens to maintain homeostatic levels of essential biometals is known to be important for survival and virulence in a host, which itself regulates metal availability as part of its response to infection. Given this importance of metal homeostasis, we sought to address how the availability of copper in particular impacts the response of the opportunistic fungal pathogen *Candida albicans* to treatment with the antifungal drug fluconazole. The present study reports whole transcriptome analysis via time-course RNA-seq of *C. albicans* cells exposed to fluconazole with and without 10 µM supplemental CuSO_4_ added to the growth medium. The results show widespread impacts of small changes in Cu availability on the transcriptional response of *C. albicans* to fluconazole. Of the 2359 genes that were differentially expressed under conditions of cotreatment, 50% were found to be driven uniquely by exposure to both Cu and fluconazole. The breadth of metabolic processes that were affected by cotreatment illuminates a fundamental intersectionality between Cu metabolism and fungal response to drug stress. More generally, these results show that seemingly minor fluctuations in Cu availability are sufficient to shift cells’ transcriptional response to drug stress. Ultimately, the findings may inform the development of new strategies that capitalize on drug-induced vulnerabilities in metal homeostasis pathways.

## Introduction

Three decades after its FDA approval, fluconazole remains a widely used antifungal against *Candida albicans*, the most common human fungal pathogen. Fluconazole is a member of the azole class of antifungals, comprised of compounds that contain an imidazole, triazole, or tetrazole group that coordinates as the sixth axial ligand to the heme iron of the fungal enzyme cytochrome P450 lanosterol 14α-demethylase (Cyp51, gene product of ERG11) (Mazu *et al.* 2016; Hargrove *et al.* 2017; Monk *et al.* 2020). Binding of azole drugs to Cyp51 blocks the biosynthesis of ergosterol, a primary component of the fungal cell membrane, thereby inhibiting cell growth (Watson *et al.* 1989; Kelly *et al.* 1995; Heimark *et al.* 2002).

Access to metal micronutrients like copper (Cu) and iron (Fe) is essential for living organisms, owing to the role of these ions in metalloprotein structure and function (Holm *et al.* 1996) as well as transition-metal signaling (Ackerman and Chang 2018). In opportunistic pathogens like *C. albicans*, metal acquisition plays a key role in virulence (Mackie *et al.* 2016; Gerwien *et al.* 2018; Li *et al.* 2019). Despite a requirement for these metals, their inherent reactivity can render them toxic if cells fail to regulate their uptake, use, detoxification, and storage to meet cells’ dynamic metal requirements irrespective of the relative metal availability in the growth environment. Regulated adaptation mechanisms include switching metal cofactors (Li *et al.* 2015; Broxton and Culotta 2016; Schatzman *et al.* 2019), activating metal uptake (Marvin *et al.* 2003, 2004; Chen *et al.* 2011; Noble 2013; Crawford *et al.* 2018), and neutralizing excess metal through storage or export (Weissman *et al.* 2000; Crawford *et al.* 2018).

Given the importance of metal homeostasis to the survival of *C. albicans*, we sought to address how the availability of transition metals, specifically Cu, impacts the outcomes of fluconazole treatment in this organism. In previous studies, we established that genes of the *C. albicans* Cu regulon are activated in response to fluconazole treatment, and supplementing the growth medium with low levels of Cu potentiates the activity of fluconazole against *C. albicans* (Hunsaker and Franz 2019a, b). Furthermore, we have shown that the level of Cu in the growth medium modulates a variety of cellular responses to fluconazole, including metal cofactor utilization, intracellular metal speciation, and production of heme (Hunsaker and Franz 2019a), establishing that small fluctuations in Cu availability can impact *C. albicans*’ adaptation to fluconazole. Our findings to date implicate a model in which *C. albicans* adaptation to fluconazole stress is hampered by Cu elevation, but the mechanistic underpinnings of this potentiation remain unclear.

In order to gain an unbiased and holistic view of how both Cu and fluconazole independently and cooperatively affect gene expression, we performed whole transcriptome analysis via time-course RNA-seq of *C. albicans* cells exposed to fluconazole under two different Cu conditions: un-supplemented YPD medium or YPD supplemented with 10 µM CuSO_4_. RNA-seq has been used to investigate the mechanism behind synergy observed for amphotericin B and lactoferrin, an Fe-chelating antimicrobial protein, and time course RNA-seq has been applied to understand the crosstalk between Cu and Zn homeostatic mechanisms in Zn-deficient *Chlamydomonas reinhardtii* cells and how transcript levels of genes involved in import and intracellular movement of Cu change over time following Zn resupply (Hong-Hermesdorf *et al.* 2014). Here, we demonstrate that Cu availability influences the *C. albicans* transcriptomic response to fluconazole stress, and co-treatment of cells with fluconazole and Cu gives rise to unique patterns of gene expression that are distinct from results obtained for individual treatment with fluconazole or Cu. Furthermore, we identify how cells reconcile opposing impacts on gene expression during co-treatment for genes in which the transcriptional response to fluconazole differs from the response to Cu.

## Materials and methods

### Yeast strains and culture conditions

Fungal stocks were maintained in 25% glycerol in YPD at −80 °C. Experiments were performed with *C. albicans* clinical isolate SC5314, which was obtained from the American Type Culture Collection (ATCC). *C. albicans* cells were streaked onto a YPD agar plate from a frozen glycerol stock and incubated at 30 °C for 24 hours. A single colony was used to inoculate liquid YPD growth medium (10 mL), and this culture was incubated overnight (∼18 hours) at 30 °C with shaking at 200 rpm. The overnight culture of *C. albicans* in YPD medium was inoculated into fresh YPD medium at an OD600 of 0.3 and allowed to grow for 3 hours at 30 °C with shaking at 200 rpm. Cultures were then either left untreated or treated with CuSO_4_ (10 µM), fluconazole (50 µM), or both CuSO_4_ and fluconazole. At the timepoints indicated in the figure legends, cells were harvested, flash frozen in liquid nitrogen, and stored at −80 °C prior to processing. YPD medium was obtained from a commercial supplier (Gibco, lot #2005064).

### Gene ontology and KEGG pathway enrichment analyses

GO term and KEGG pathway enrichment analyses were carried out for each cluster at each time point by using Chi-Square Test with *P*-value threshold 0.05. The gene list of GO terms was acquired from Candida Genome Database (http://www.candidagenome.org/download/go/go_slim/) (Skrzypek *et al.* 2017). KEGG pathway gene lists were retrieved from the KEGG database (https://www.genome.jp/kegg/kegg1.html) (Kanehisa and Goto 2000; Kanehisa *et al.* 2004). GO Trim terms were created manually by forming logical groupings of existing GO terms related to metal homeostasis categories of interest. Not all GO terms were assigned to a GO Trim term and some GO terms were assigned to more than one GO Trim term. A complete list of GO Trim terms and their associated GO terms and genes is provided in Supplemental File 6.

Additional Materials and Methods are contained in Supplemental File S1.

## Results and discussion

### Overview of changes to the transcriptome

**Figure 1.**
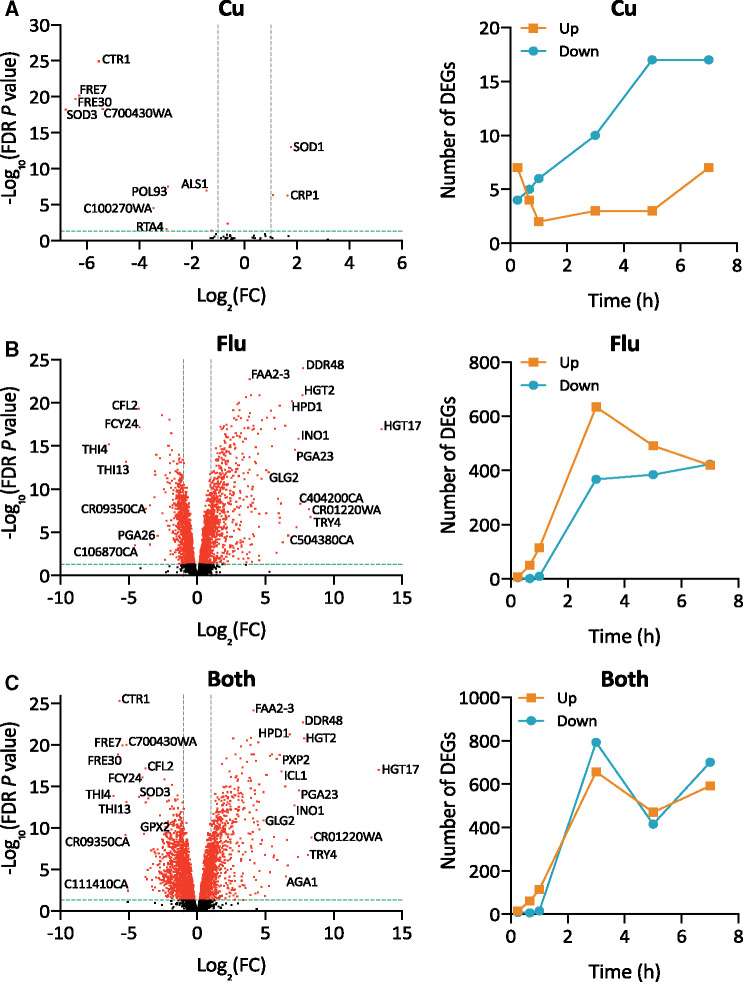
Differentially expressed genes (DEGs) following treatment with Cu (A), fluconazole (Flu, B), or Both (C). Left panel: Volcano plots of DEGs at 3 hours. Dashed green lines indicate the cut-off for FDR *P*-values <0.05 and dashed gray lines indicate boundaries for Log_2_(FC) ± 1. Points with FDR *P*-values <0.05 are red and those >0.05 are black. Genes falling above the dashed green line and outside of the dashed gray lines were considered significantly changed. Right panel: Number of significant DEGs following treatment with Cu, fluconazole, or Both over time. Upregulated genes are indicated with orange squares and downregulated genes are indicated with teal circles. Significant DEGs had a log FC ≥1 or ≤−1 and an FDR *P*-value <0.05.

*C. albicans* cells were treated with 50 µM fluconazole, 10 µM CuSO_4_, or both fluconazole and Cu (hereafter referred to as “Both”) in YPD medium (containing approximately 0.14 µM basal Cu as determined by ICP-MS analysis, Supplementary Table S1) and harvested after 0.25, 0.67, 1, 3, 5, and 7 hours. These concentrations were chosen based on results from our prior studies to ensure a robust transcriptional response from fluconazole in a culture with relatively high cell density but without fully inhibiting cell growth; 10 µM Cu was chosen as we previously found it to be sufficient to modulate fluconazole efficacy while being well below the 25 mM MIC of Cu toxicity under these conditions (Hunsaker and Franz 2019a, b). Differentially expressed genes (DEGs) with log fold changes ≥1 or ≤−1 and false discovery rate (FDR) *P*-values <0.05 were considered to be significantly changed. Compared to other treatments, cells treated only with Cu had a low number of DEGs (Figure 1A). Over 7 hours of Cu treatment, the total number of significantly downregulated genes increased from 4 to 17, while the number of upregulated genes remained between 2 and 7, relative to a separate untreated control (Figure 1A). The low numbers of genes altered by exposure to Cu bolsters the conclusion that this concentration of Cu is not sufficient to induce significant stress on its own. By contrast, after 3 hours of fluconazole treatment, the transcriptome had 634 and 367 genes with a significant increase or decrease in expression, respectively, relative to control cells (Figure 1B). At the same timepoint, the transcriptome of cells treated with Both had 657 and 793 genes induced and repressed, respectively, relative to untreated controls (Figure 1C). Volcano plots illustrate the minimal impact of low micromolar supplemental Cu on the transcriptome compared to that of fluconazole (Figure 1, Supplementary Figure S1). Full gene lists with log fold changes and *P* values for all conditions and timepoints are provided in Supplemental File S2.

### Relating the effects of Cu and fluconazole: clusters and drivers of gene expression

The central objective of this study was to address how a small increase in environmental Cu levels influences the transcriptomic response of *C. albicans* during fluconazole treatment of cells in culture. We hypothesized that the expression of some, potentially many, genes is differently impacted by individual treatment with fluconazole or Cu and sought to determine how cells reconcile these conflicting transcriptional responses during co-treatment with both fluconazole and Cu. We further posited that fluconazole treatment may compromise the ability of *C. albicans* to manage excess Cu, and similarly, even mild Cu elevation may interfere with *C. albicans*’ response to fluconazole.

The response of individual genes was coded by a 3-number pattern corresponding to the effect of the treatment condition: −1, 0, 1 for repression, no change, or induction, respectively. Mathematically, for three treatments (Cu, fluconazole, or Both) and three expression outcomes (repression, no change, or induction), there are 27 possible number combinations, or expression patterns, 17 of which appeared in the data and were termed “clusters” (Table 1). Genes were placed into clusters based on their expression pattern at each timepoint. Each gene can only belong to one cluster at a given timepoint, but the cluster to which a gene belongs may change over time. Tables of all the significant DEGs at each timepoint and their designated clusters are provided in Supplemental File S3.

**Table 1:** **Description of clusters.** Expression pattern coding: −1 (repression), 0 (no change), 1 (induction).

Expression Pattern (Cu, Flu, Both)	Cluster ID #	Type	Driver of Both	Description
−1, −1 ,−1	1	D	Cu, Flu	Repression driven by Cu and Flu
−1, −1, 0	2	C	Both	Cu and Flu each repress; no sig. change for Both
−1, −1, 1	—	—	—	(not present in the data)
−1, 0, −1	3	B	Cu	Repression driven by Cu
−1, 0, 0	4	A	Flu	Repressed by Cu; Flu prevents repression for Both
−1, 0, 1				(Not present in the data)
−1, 1, −1	5	A	Cu	Repression by Cu overrides induction by Flu
−1, 1, 0	6	D	Both	Repression by Cu and induction by Flu cancel out
−1, 1, 1	7	A	Flu	Repression by Cu overridden by induction by Flu
0, −1, −1	8	B	Flu	Repression driven by Flu
0, −1, 0	9	A	Cu	Repressed by Flu; Cu prevents repression for Both
0, −1, 1	—	—	—	(Not present in the data)
0, 0, −1	10	C	Both	Repressed uniquely by Both
0, 0, 0				(Not present in the data)
0, 0, 1	11	C	Both	Induced uniquely by Both
0,1,−1	—	—	—	(Not present in the data)
0, 1, 0	12	A	Cu	Induced by Flu; Cu prevents induction for Both
0, 1, 1	13	B	Flu	Induction driven by Flu
1, −1, −1	14	A	Flu	Repression by Flu overrides induction by Cu
1, −1, 0	15	D	Both	Repression by Flu and induction by Cu cancel out
1, −1, 1	—	—	—	(Not present in the data)
1, 0, −1	—	—	—	(Not present in the data)
1, 0, 0	16	A	Flu	Induced by Cu; Flu prevents induction for Both
1, 0, 1	17	B	Cu	Induction driven by Cu
1, 1, −1	—	—	—	(Not present in the data)
1, 1, 0	—	—	—	(Not present in the data)
1, 1, 1	—	—	—	(Not present in the data)

When cells received a single treatment, any changes to gene expression can be attributed to that treatment alone, making it the driver of gene expression by default. However, during treatment with Both, the effect of either Cu or fluconazole may dominate. The driver of expression during treatment with Both was therefore identified as the condition for which its digit matched that of the third digit in the expression pattern (the digit representing Both). When the first digit matches the third, Cu is the driver; when the second digit matches the third, fluconazole is the driver. When neither the first nor the second match the third digit, Both is the driver. Interestingly, we found many instances in which treatment with Both uniquely impacted gene expression such that the observed effect did not reflect that of either fluconazole or Cu individually. These examples reveal that Cu and fluconazole in combination generate unique transcriptional responses. To relate the impacts of individual treatment with Cu or fluconazole to impacts of treatment with Both, each of the 17 clusters was categorized into one of four types, A–D (Table 1).

### Cluster Type A: Overrider

Clusters in which the effect of fluconazole overrode the effect of Cu or the effect of Cu overrode the effect of fluconazole during treatment with Both were designated Type A clusters. In these clusters, the individual impacts of treatment with fluconazole or Cu on gene expression were significant but different, and one of these impacts dominated the outcome of treatment with Both. Clusters 4, 5, 7, 9, 12, 14, and 16 were classified as Type A clusters. Cu drove the response in Clusters 5, 9, and 12, and fluconazole drove the response in Clusters 4, 7, 14, and 16.

### Cluster Type B: Bystander

In some cases, only one treatment significantly impacted gene expression and that same impact was reflected in the response to treatment with Both. These clusters (3, 8, 13, and 17) were classified as Type B. In Clusters 3 and 17, Cu but not fluconazole caused a significant change in expression. Treatment with Both reflected the impact of Cu alone, suggesting that fluconazole was a “bystander,” and the response to Both stemmed from Cu. Conversely, in Clusters 8 and 13, fluconazole but not Cu caused a significant change in expression and Both reflected the effects of fluconazole, making Cu the bystander.

### Cluster Type C: Collaborator

There were three clusters (2, 10, and 11) in which treatment with Both impacted gene expression in a way that did not match either of the individual impacts of Cu or fluconazole, indicating that the combination of these two treatments had a unique effect on gene expression. The expression patterns of Clusters 10 and 11 are 0, 0, −1 and 0, 0, 1, respectively, indicating that fluconazole and Cu did not significantly impact gene expression individually, but treatment with Both caused repression or induction. In the third Type C cluster, Cluster 2, Cu and fluconazole each repressed expression, but Both caused no significant change.

### Cluster Type D: Other

Clusters 1, 6, and 15 did not fall neatly into the first three types of clusters and were classified as other, Type D clusters. For genes falling into Cluster 1, repression was observed for fluconazole, Cu, and Both, meaning fluconazole and Cu were both drivers. In Clusters 6 and 15, there was no significant change in gene expression for Both, seemingly due to repression and induction by the individual treatments cancelling out.

### Prevalence and magnitude of top DEGs in clusters

**Figure 2 .**
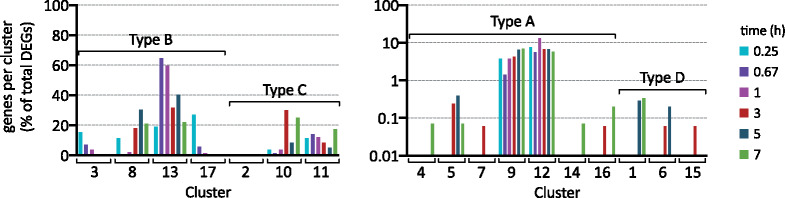
Prevalence of each cluster over time. Percent of DEGs assigned to each cluster after 0.25 (teal), 0.67 (purple), 1 (pink), 3 (red), 5 (navy), and 7 (green) h of treatment. Types B and C clusters are shown in the plot on the left on a linear scale. Types A and D clusters are shown in the plot on the right on a log 10 scale.

The frequency with which each cluster appears in the data reveals how Cu and fluconazole each drive the *C. albicans* transcriptome during treatment with Both. To determine which clusters are most highly represented in the dataset, the percentage of DEGs falling into each cluster was calculated for each timepoint (Figure 2). Types A and D clusters were less common than Types B and C, meaning that genes were most likely either to be driven by a single treatment or be uniquely driven by Both. Genes in most of the Types A and D clusters comprised only 0–0.4% of all DEGs at any timepoint, with the exception of Clusters 9 and 12, which represented up to 7–14% of DEGs at some timepoints. By comparison, Types B and C clusters captured up to 30 and 65% of DEGs at some timepoints, respectively. Furthermore, ranking the Top DEGs (log fold change ≥2 or ≤−2 with an FDR *P*-value <0.05) revealed that the majority of genes that are most significantly changed in magnitude follow this same pattern, with Type B Clusters 13 and 8 (fluconazole-driven, Cu-bystander induction, and repression, respectively) accounting for 70% of 1798 cluster occurrences among the Top DEGs, followed by “collaborator” Type C Clusters 10 and 11 (repression and induction respectively driven by Both) being the second-most prevalent (Supplemental File S2).

Cu-driven repression (Cluster 3) and induction (Cluster 17) were most highly represented at 0.25 hours and then decreased over time, consistent with cells reestablishing Cu homeostasis early. This decrease in Cu-driven changes coincides with an increase in the total number of DEGs over time. The percentage of genes that were uniquely repressed by Both (Cluster 10) increased at 3 and 7 hours, whereas the percentage uniquely induced by Both (Cluster 11) decreased steadily between 0.67 and 5 hours with a sharp increase at 7 hours. The total number of clusters represented in the dataset grew over time from seven at 0.67 hours to fourteen at 7 hours (Supplementary Figure S2), revealing a progressive increase in the complexity of the impacts on the transcriptome and illustrating the value of analyzing data across multiple timepoints.

**Figure 3.**
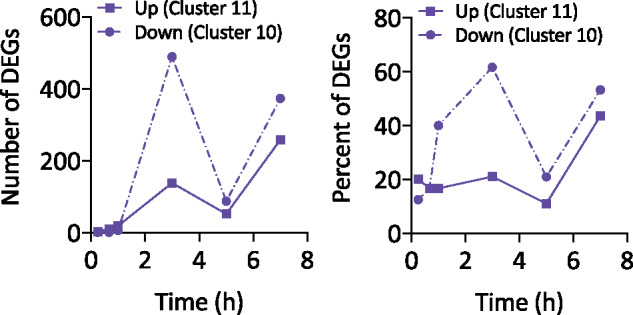
Total number (A) and percentage (B) of DEGs significantly upregulated (circles with dashed line) or downregulated (squares with solid line) only during treatment with Both.

Taken together, an analysis of cluster prevalence revealed that it was more common for a single treatment to determine the impact of Both on gene expression (Type B) or for Both to have a unique impact on gene expression (Type C). Strikingly, of the genes differentially expressed after 3 hours of treatment with Both, 138 and 489 genes were uniquely induced and repressed, respectively (Figure 3A), meaning that treatment with Cu or fluconazole alone had no significant impact on their expression, but treatment with Both did have a significant impact. These numbers equate to approximately 60% of the induced genes and 21% of the repressed genes that uniquely responded to treatment with Both at 3 hours (Figure 3B). The high prevalence of Clusters 10 and 11 reveals that Cu availability has a substantial influence on the transcriptomic response of *C. albicans* to fluconazole treatment.

### Functional analysis

To determine the biological processes and pathways associated with treatment with Cu, fluconazole, and Both, the lists of genes associated with each cluster at each timepoint were separately analyzed for enrichment of gene ontology (GO; Ashburner *et al.* 2000; The Gene Ontology Consortium 2018) and Kyoto Encyclopedia of Genes and Genomes (KEGG; Kanehisa *et al.* 2004) terms. In our enrichment tests, we found that the 6,149 GO terms to which *C. albicans* genes are assigned were too specific to generate significant enrichment results. Instead, higher-level GO Slim terms that group genes into broader categories were used. Clusters 10 and 11 were selected as the primary focus for functional analysis to provide insight regarding the biological processes specifically impacted by cotreatment with Both. We focused on data from 3 to 7 hours because there were more DEGs at these timepoints and therefore more significantly enriched terms relative to earlier timepoints. Given that the genes associated with a cluster could change over time, the GO and KEGG terms enriched in a cluster could change over time as well. Significant results from the GO and KEGG enrichment tests for all clusters at all timepoints are provided in Supplemental Files S4 and S5.

### GO enrichment analysis

**Figure 4.**
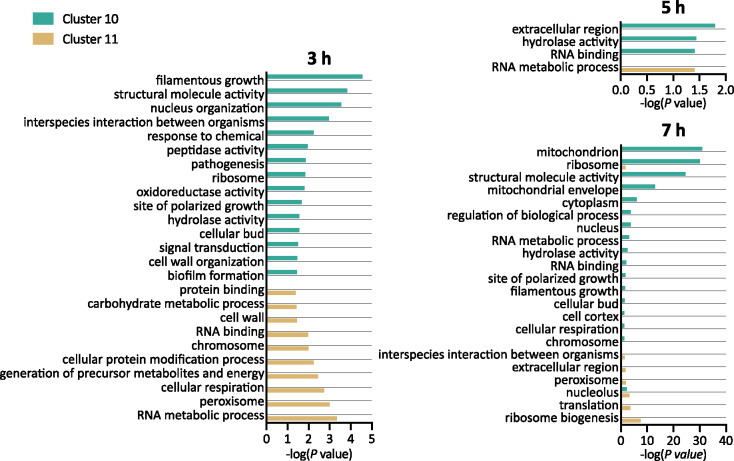
GO analysis of genes differentially expressed by Both, but not by fluconazole or Cu alone. Functional enrichment of genes significantly repressed (Cluster 10) or induced (Cluster 11) uniquely by Both after 3, 5, and 7 hours of treatment. Significantly enriched GO Slim terms (*P*-value <0.05) are listed on the *y*-axis, and the negative log of the *P-*value (base 10) is indicated on the *x*-axis.

The GO Slim terms most significantly enriched for genes uniquely repressed by Both (Cluster 10) at 3 hours included filamentous growth, structural molecule activity, and nucleus organization (Figure 4). At 5 hours, only three terms were significantly enriched: extracellular region, hydrolase activity, and RNA binding. Significantly enriched terms for genes that were repressed at 7 hours included mitochondrion, ribosome, structural molecule activity, and mitochondrial envelope. Genes that were induced uniquely by Both (Cluster 11) were most significantly enriched for RNA metabolic process, peroxisome, and cellular respiration. Cluster 11 genes continued to be enriched for the term RNA metabolic process at 5 hours, and at 7 hours, ribosome biogenesis and translation were enriched.

### KEGG enrichment analysis

**Figure 5.**
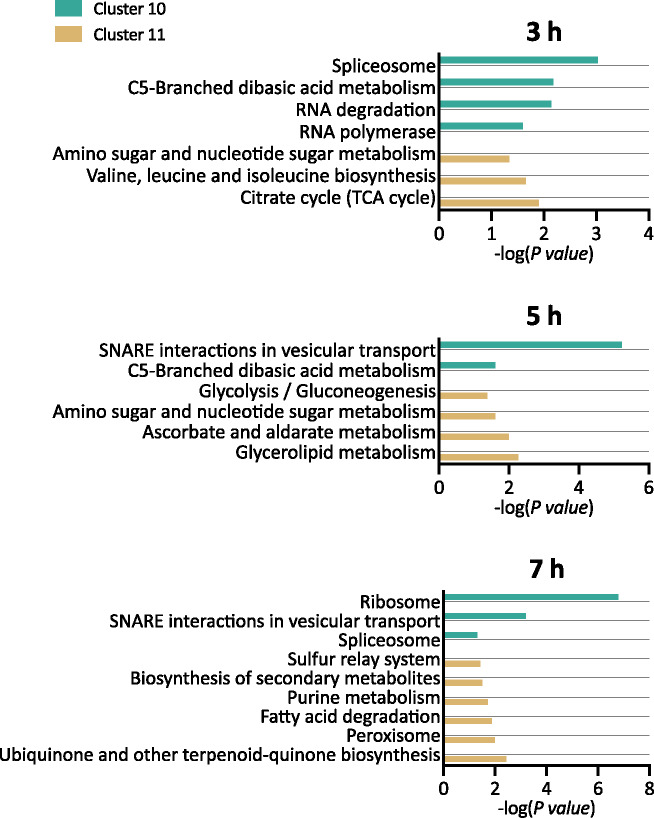
KEGG analysis of genes differentially expressed by Both, but not by fluconazole or Cu alone. Functional enrichment of genes significantly repressed (Cluster 10) or induced (Cluster 11) uniquely by Both after 3, 5, and 7 hours of treatment. Significantly enriched KEGG terms (*P*-value <0.05) are listed on the *y*-axis, and the negative log of the *P-*value (base 10) is indicated on the *x*-axis. Superscripts designate terms enriched in other clusters: ^#^8, ^^^9, ^+^12, *13.

Genes that were uniquely repressed (Cluster 10) or induced (Cluster 11) by Both at 3 hours were enriched for terms related to carbohydrate and amino acid metabolism, transcription, and RNA degradation (Figure 5, see Supplemental File S5 for list of genes represented in the data for each term). Carbohydrate metabolism was also impacted by Both at 5 hours, with Clusters 10 and 11 enriched for several terms under this category, as well as terms related to lipid metabolism and vesicular transport. The cellular impacts at 7 hours related to transcription, translation, nucleotide metabolism, lipid metabolism, and other metabolic processes. Nearly half of the 22 KEGG terms enriched in Clusters 10 and 11 were also significantly enriched in at least one other cluster at the same timepoint (Figure 5). For example, Cluster 11 (induction by Both only) and Cluster 13 (induction by fluconazole and by Both) were each enriched at 3 hours for genes involved in the citrate cycle. However, although some clusters are enriched for the same KEGG terms, there is no overlap in the DEGs that give rise to the enrichment, since a gene can only belong to one cluster at a time. Taken together, the KEGG analysis reveals that co-treatment with fluconazole and Cu impacts several pathways that the individual treatments do not, while also expanding the list of genes contributing to enrichment in already impacted pathways.

### Metal-focused “GO Trim” enrichment analysis

**Figure 6.**
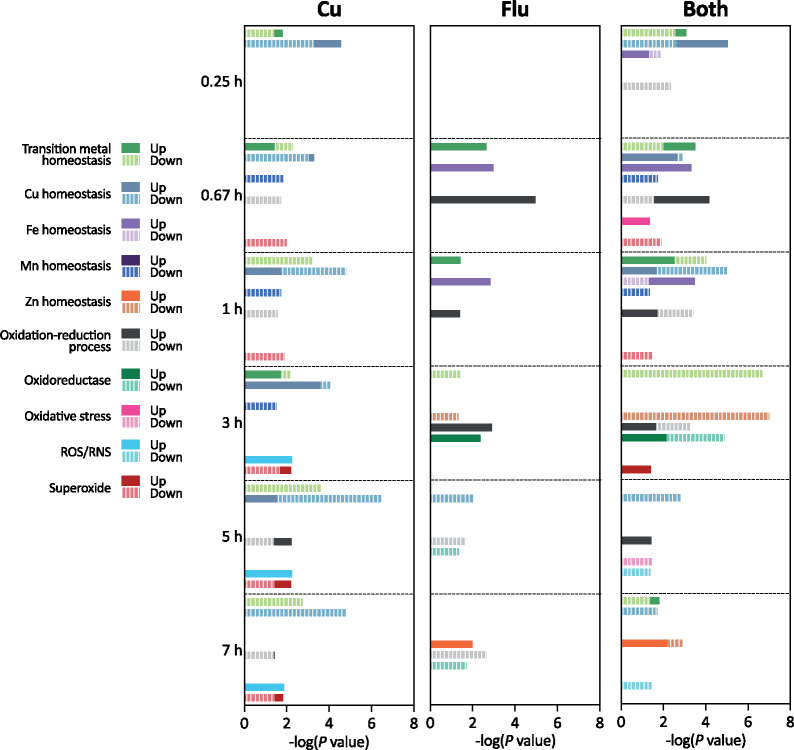
Metal-focused GO Trim analysis of DEGs following treatment with Cu, fluconazole (Flu), or Both at 0.25, 0.67, 1, 3, 5, or 7 hours. For each timepoint, GO Trim terms are plotted in the order they are listed in the legend. Blanks indicate no term enrichment for a given condition or timepoint. Up- (solid bars) or down-regulation (striped bars) are indicated for each GO Trim term. If a term was enriched for both up- and down-regulated genes, the bars are superimposed on the graph.

Neither GO Slim nor KEGG databases contain terms explicitly related to metal homeostasis; therefore, to probe the enrichment of genes along this dimension, we defined “GO Trim” terms by grouping specific GO terms under more general GO Trim categories (Supplemental File S6**)**. For example, 14 unique GO terms related to Cu transport and utilization were combined under a customized GO Trim term called “Cu homeostasis.” As shown in Figure 6, the lists of DEGs for each of the three treatments were enriched for genes involved in transition metal homeostasis at nearly all timepoints. Full GO Trim enrichment data, including additional terms not plotted in Figure 6, is available in Supplemental File S7. Notably, fluconazole alone leads to upregulation of transition metal homeostasis generally, Fe homeostasis particularly, and oxidation-reduction processes across several timepoints. Treatment with Both seems to confuse this response by downregulating several genes associated with these processes, or further exaggerating the fluconazole response (as in downregulation of Zn homeostasis at 3 hours). There were also several terms that were only enriched among genes significantly impacted by Both: Fe homeostasis and oxidation-reduction process at 0.25 hours, oxidative stress at 0.67 and 5 hours. This analysis demonstrates that impacts of drug treatment on metal-associated cellular processes can be influenced by the Cu content in their environment.

### Targeted analysis of genes involved in metal homeostasis and ergosterol biosynthesis

To complement the analyses performed at the cluster level, time-course expression profiles were compared for cells treated with Cu, fluconazole, or Both for handpicked genes of interest. Genes related to Cu, Fe, Mn, and Zn homeostasis were chosen due to our overarching interest in the effect of antifungal treatment on regulation of d-block biometals. Genes related to ergosterol and heme biosynthesis were chosen because fluconazole interferes with ergosterol biosynthesis via inhibition of a heme-dependent enzyme. In each of these cases, we were also interested in the intersectional impact of Cu availability on these processes at the transcript level.

### Copper Homeostasis

**Figure 7.**
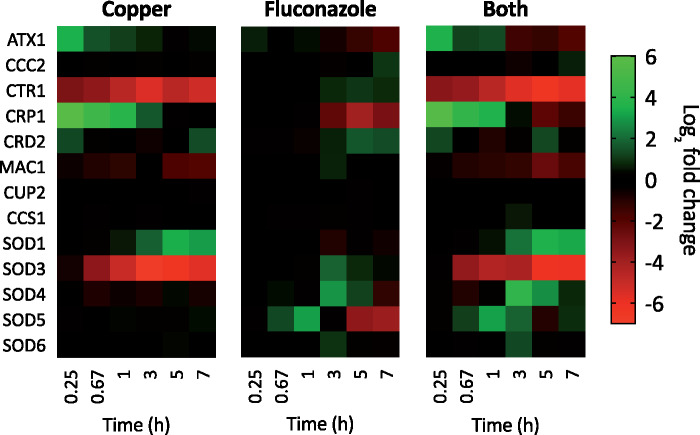
Heat maps showing log_2_ fold change expression for genes involved in Cu homeostasis. Genes are indicated on the left and timepoints (0.25, 0.67, 1, 3, 5, or 7 hours) are shown at the bottom of each map. Data are normalized to mRNA levels of untreated cells. Conditions: (Flu) = 50 µM, (CuSO_4_) = 10 µM in YPD medium. All log fold changes are shown, regardless of their statistical significance.

Results from the present study are consistent with and expand upon our earlier work showing differential expression of key genes of the Cu regulon (Hunsaker and Franz 2019a). As shown in Figure 7, fluconazole and Cu have opposing effects on many of these genes. Cu importer *CTR1* was strongly repressed by Cu and Both, but induced by fluconazole after 3 hours. The Cu-binding metallothionein *CRD2* was also induced by fluconazole. *C. albicans’* second metallothionein *CUP1* did not appear in the sequencing data, but we have previously found its transcript levels elevated in response to fluconazole (Hunsaker and Franz 2019a). Cu exporter *CRP1* was strongly induced early by Cu and progressively returned to basal levels. By contrast, *CRP1* was repressed by fluconazole and by Both at later timepoints.

Atx1 is a chaperone that delivers Cu to Ccc2, a P-type ATPase of the Golgi, where the multicopper oxidase (MCO) Fet3 is metalated (Chen *et al.* 2011). Expression of *ATX1* was initially induced by Cu then progressively returned to baseline, whereas fluconazole did not impact *ATX1* early but caused repression later. Treatment with Both gave rise to a mix of these two responses, reminiscent of the expression pattern for *CRP1* in which Cu appeared to drive the early induction response, with fluconazole taking over and repressing at later timepoints. Expression of *CCC2* was not significantly impacted by the treatments, though slight induction was evident at 7 hours with fluconazole or Both. Expression of *CUP2*, the transcription factor involved in the response to high Cu, was not impacted by any of the three treatments, whereas the Cu-sensing transcription factor *MAC1* that allows *C. albicans* to respond to low Cu was repressed by Cu and by Both but slightly induced by fluconazole at 3 hours, correlating with the timing of fluconazole’s induction of *CTR1*, which is regulated by Mac1.

*C. albicans* has six superoxide dismutase (SOD) enzymes: cytosolic Cu/Zn-Sod1, mitochondrial Mn-Sod2, cytosolic Mn-Sod3, and Cu-only Sod4, Sod5, and Sod6 that reside on the cell surface. With the exception of *SOD1* and *SOD6*, *SOD* transcripts were all significantly impacted by fluconazole. Although Sod3 utilizes Mn as a cofactor, the switch from Sod1 to Sod3 is understood to occur as a result of Cu limitation, not Mn availability (Li *et al.* 2015). The moderate repression of *SOD1* and significant induction of *SOD3* in our data by fluconazole alone is therefore symptomatic of a functional Cu deficiency in *C. albicans* under these conditions, as we have previously observed (Hunsaker and Franz 2019a). Cu supplementation overrode fluconazole’s effect, resulting in strong induction of *SOD1* and repression of *SOD3*. Expression of the Cu chaperone for Sod1 (*CCS1*) was not significantly impacted by any of the treatments. *SOD4* transcripts increased upon fluconazole treatment, and even more so with Both. *C. albicans* has been shown to induce *SOD4* in response to Fe starvation (Schatzman *et al.* 2019), and we have previously demonstrated that fluconazole treatment depletes EPR-detectable labile Fe pools, despite a twofold increase in bulk cellular Fe (Hunsaker and Franz 2019a). These combined findings are consistent with fluconazole-induced biological Fe starvation, despite chemical Fe sufficiency. Sod5, which acquires its Cu extracellularly as opposed to through the secretory pathway (Gleason *et al.* 2014), appears to be important in *C. albicans* pathogenesis, as its deletion eradicated *C. albicans* biofilm in a rat intravenous catheter model. It is therefore interesting that fluconazole treatment resulted in a progressive increase in *SOD5* expression during the first hour followed by repression at 5 and 7 hours with the inversion occurring at 3 hours. Treatment with Both induced *SOD5* from 0.67 to 3 hours but had no significant effect on expression at 5 and 7 hours, differing from the response of fluconazole alone. Together, these results show that fluconazole impacts the suite of *SOD* transcripts in ways that can be swayed by access to Cu.

### Iron homeostasis

**Figure 8.**
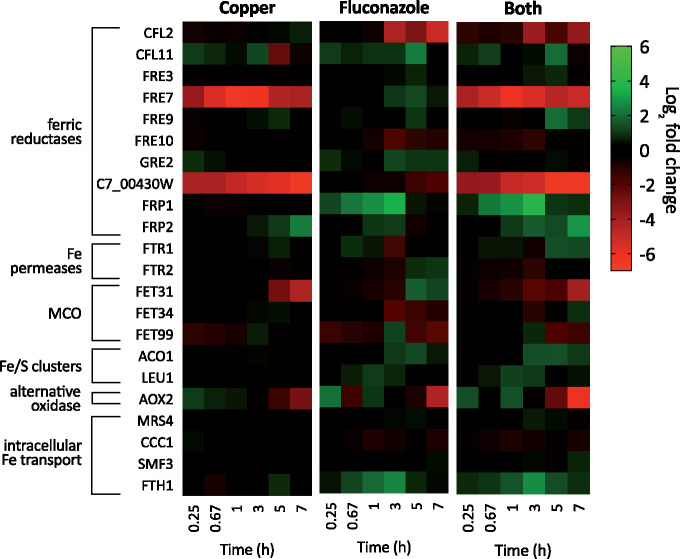
Heat maps showing log_2_ fold change expression for genes involved in Fe homeostasis. Genes are indicated on the left and timepoints (0.25, 0.67, 1, 3, 5, or 7 hours) are shown at the bottom of each map. Data are normalized to mRNA levels of untreated cells. Conditions: (Flu) = 50 µM, (CuSO_4_) = 10 µM in YPD medium. All log fold changes are shown, regardless of their statistical significance. MCO, MCO.

In *C. albicans*, Fe uptake occurs via reduction of Fe(III) to the more soluble Fe(II) by cell surface ferric reductases, followed by oxidation and internalization mediated by a MCO–Fe permease complex (Chen *et al.* 2011). *C. albicans* has as many as 17 ferric reductase-like genes, though it is unlikely all of them actually function as ferric reductases (Fourie *et al.* 2018). Cu and fluconazole each caused differential expression of several of these reductases, and the response to Both often consisted of a mixture of the responses to the individual treatments. Cu supplementation most strongly repressed the expression of ferric reductases *FRE7* and *C7_00430W*, regardless of fluconazole treatment (Figure 8). Fluconazole treatment induced several ferric reductases, most notably *FRP1* and *FRE7*, and repressed others, including *CFL2*, *FRE10*, and *C7_00430W*. Treatment with Both repressed *FRE7* and *C7_00430W*, presumably driven by Cu, and *CFL2*, presumably driven by fluconazole. *FRP1* and *FRP2* were induced by Both, consistent with fluconazole’s induction of *FRP1* and Cu’s induction of *FRP2*.

High affinity Fe import occurs through cell membrane Fe permeases *FTR1* and *FTR2*. Expression of these genes was not strongly impacted by Cu, but fluconazole treatment caused differential expression of both genes, regulating them in an inverse manner over time (Figure 8). Treatment with Both had a unique effect on expression of *FTR1* and *FTR2*: *FTR1* was induced at almost every timepoint, except at 3 hours when it was repressed, while *FTR2* was mostly repressed and never induced. The MCO *FET31* was repressed by Cu from 5 to 7 hours but induced by fluconazole during the same period (Figure 8). Interestingly, treatment with Both resulted in repression at every timepoint after 15 m, indicating Cu has a stronger influence at later timepoints. The impacts to two other MCOs, *FET34* and *FET99* were more subtle. Transcript levels of *ACO1* and *LEU1*, which encode iron-sulfur containing proteins, were not impacted by Cu, but they were induced by fluconazole at several timepoints. Treatment with Both resulted in an expression pattern very similar to that of fluconazole, suggesting fluconazole primarily drives expression of these genes.

Intracellular Fe homeostasis in *C. albicans* is largely mediated the Mrs4-Ccc1-Smf3 pathway (Xu *et al.* 2014). Mrs4 is a mitochondrial Fe importer, and Ccc1 and Smf3 are vacuolar Fe transporters that promote vacuolar Fe storage and export, respectively. Despite our previous observation that fluconazole treatment depletes the labile Fe pool detectable by whole-cell electron paramagnetic resonance (Hunsaker and Franz 2019a), significant differential expression of *MRS4*, *CCC1*, or *SMF3* was not detected by RNA-seq (Figure 8), as may be expected upon the change in Fe localization and speciation. However, expression of *FTH1*, a high affinity Fe transporter for intravacuolar stores of Fe, was induced upon fluconazole treatment with or without Cu supplementation, suggesting that *C. albicans* may recruit vacuolar Fe stores in response to fluconazole stress. Recruitment of vacuolar Fe is consistent with an increased need for this metal to support the synthesis of Fe-containing proteins, including but not limited to Cyp51, the heme-containing target of fluconazole.

### Manganese homeostasis

**Figure 9.**
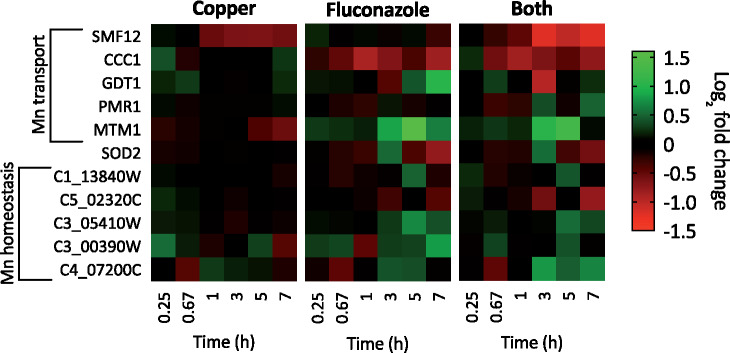
Heat maps showing log_2_ fold change expression for genes involved in Mn homeostasis. Genes are indicated on the left and timepoints (0.25, 0.67, 1, 3, 5, or 7 hours) are shown at the bottom of each map. Data are normalized to mRNA levels of untreated cells. Conditions: (Flu) = 50 µM, (CuSO_4_) = 10 µM in YPD medium. All log fold changes are shown, regardless of their statistical significance.

Several genes in the *C. albicans* genome have been annotated as having a role in Mn homeostasis, primarily based on orthologous genes in *S. cerevisiae* (Reddi *et al.* 2009). Genes involved in Mn homeostasis had an overall lower change in expression relative to other metal regulons in response to treatment with Cu, fluconazole, or Both. *SMF12* is a Mn transporter that is an ortholog of *S. cerevisiae* *SMF1*. Smf1 localizes to the cell surface during Mn starvation but is directed to the vacuole for degradation under Mn replete conditions (Liu and Culotta 1999; Portnoy *et al.* 2000). However, Smf proteins can be promiscuous in the metal ions they transport, and Smf1 has been shown to contribute to cellular accumulation of cadmium and Cu. A potential role in low affinity Cu transport could explain why *SMF12* is downregulated by Cu and even more downregulated by Both (Figure 9).

The vacuolar Fe transporter *CCC1* is also believed to transport Mn ions. Repression of *CCC1* was evident in cells treated with fluconazole or Both (Figure 9), a finding in line with *C. albicans* reducing storage of Fe and/or Mn during adaptation to fluconazole stress. PMR1 is a secretory pathway P-type ATPase that transports Ca(II) and Mn(II) ions to the Golgi. *GDT1* is also a transmembrane Ca(II) and Mn(II) transporter that supports the function of *PMR1* for Ca and Mn transport into the Golgi. Although neither of these genes were strongly impacted under our conditions, repression of GDT1 by Both was evident at 3 hours and induction of *GDT1* by fluconazole occurred at 7 hours (Figure 9).

In *S. cerevisiae*, *MTM1* is a crucial component for Sod2 activation in mitochondria and thought to function as a Mn chaperone for Sod2. Interestingly, *MTM1* was induced by fluconazole and by Both at several timepoints, most strongly at 5 hours. Additional genes thought to be involved in Mn homeostasis but for which exact roles are not known also showed some changes in expression (Figure 9).

### Zinc Homeostasis

**Figure 10.**
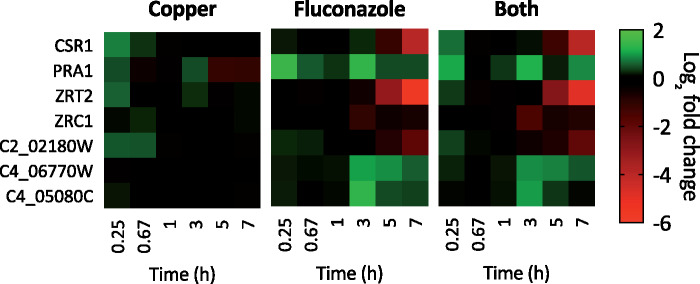
Heat maps showing log_2_ fold change expression for genes involved in Zn homeostasis. Genes are indicated on the left and timepoints (0.25, 0.67, 1, 3, 5, or 7 hours) are shown at the bottom of each map. Data are normalized to mRNA levels of untreated cells. Conditions: (Flu) = 50 µM, (CuSO_4_) = 10 µM in YPD medium. All log fold changes are shown, regardless of their statistical significance.

*C. albicans* has two plasma membrane zinc importers, Zrt1 and Zrt2, which are positively regulated by transcription factor *CSR1* (also known as *ZAP1*) and expressed under neutral and acidic pH, respectively. *CSR1* was repressed by fluconazole and by Both at 5 hours and even more repressed at 7 hours (Figure 10). *ZRT2* was also repressed from 5 to 7 hours, regardless of Cu supplementation.

In addition to the expression of membrane transporters, *C. albicans* secretes a Zn-binding protein called Pra1 that scavenges extracellular Zn and delivers it to the fungal cell via Zrt1 (Citiulo *et al.* 2012). Interestingly, although *ZRT2* was repressed by fluconazole, *PRA1* was induced by fluconazole and by Both at several timepoints (Figure 10), suggesting *C. albicans* relies more heavily on Zn scavenging versus direct import under these conditions. Following import, Zn is stored in vesicular compartments termed “zincosomes,” a process essential for tolerance to Zn that is mediated by Zn transporter Zrc1 (C2_02200W) (Crawford *et al.* 2018). *ZRC1* was slightly repressed by fluconazole and by Both at 3 hours. *C2_02180W* is an ortholog of *S. cerevisiae* *ZRT3*, a vacuolar Zn transporter that mobilizes stored Zn for use in the cytosol during Zn deficiency. Its expression was not significantly impacted by any of the three treatment conditions. *C4_06770W* and *C4_05080C* are predicted Zn transporters localized to the endoplasmic reticulum. Interestingly, they are each induced by fluconazole and by Both beginning at 3 hours.

### Ergosterol biosynthesis

**Figure 11.**
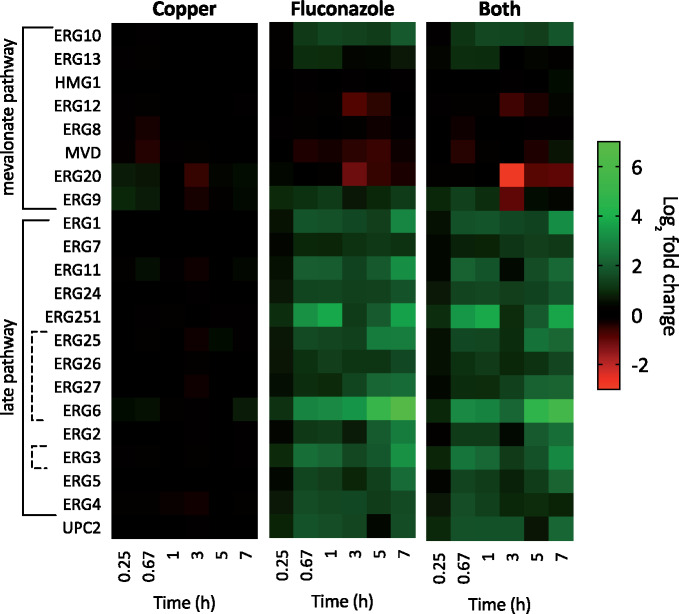
Heat maps showing log_2_ fold change expression for genes involved in ergosterol biosynthesis after 0.25, 0.67, 1, 3, 5, or 7 hours of treatment. Genes are listed in order as they appear in the pathway. Genes involved in the alternate pathway are indicated by dashed lines. UPC2 is the transcription factor that activates sterol biosynthesis genes. Data are normalized to mRNA levels of untreated cells. Conditions: (Flu) = 50 µM, (CuSO_4_) = 10 µM in YPD medium.

Ergosterol biosynthesis is a 25-step process consisting of three pathways: mevalonate, late, and alternate (Bhattacharya *et al.* 2018). Inhibition of Cyp51 (gene product of *ERG11*) by azoles activates a split from the late pathway to the alternate pathway, resulting in the synthesis of alternate sterols that allow *C. albicans* to survive in the absence of ergosterol. The genes involved in the alternate pathway are indicated by a dashed line in Figure 11.

There is an established role for Cu in lipid metabolism, and treatment with millimolar levels of Cu has been shown to increase flux through the ergosterol biosynthetic pathway of yeast, resulting in significantly higher levels of ergosterol and its precursors that suppress the effectiveness of statins. Statins inhibit 3-hydroxy-3-methylglutaryl (HMG)-CoA reductase (gene product of *HMG1*) (Maciejak *et al.* 2013), an enzyme upstream of lanosterol 14α-demethylase (*ERG11*) in the ergosterol biosynthetic pathway. The same report illustrated that supplementation with 1–2 mM CuSO_4_ or ZnSO_4_ rescued growth of ketoconazole-treated cells (Fowler *et al.* 2011), a finding that contrasts with our discovery that ketoconazole-treated *C. albicans* cells have reduced growth when supplemented with 100 µM Cu (Hunsaker and Franz 2019b). However, the statin experiments were performed in *S. cerevisiae*, not *C. albicans*, and much higher Cu concentrations were used. Furthermore, Cu supplementation was performed after statin treatment, rather than concurrently as in our experiments.

Given the link between Cu and lipid metabolism, we wondered if Cu may influence ergosterol biosynthesis in ways that impact *C. albicans’* recovery from fluconazole treatment. As shown in Figure 11, this level of Cu alone did not impact the expression of genes in this pathway, while fluconazole did, as expected. The most highly induced gene was *ERG6*, which mediates the transition from the late to the alternate pathway during fluconazole stress, suggesting this adaptation mechanism occurs under our conditions. In general, supplementation with Cu did not change the expression of these genes relative to fluconazole alone, indicating that this modest level of Cu does not impact flux through the ergosterol biosynthetic pathway at the transcriptional level. Interestingly, however, a transcriptome study of *C. albicans* under much higher levels of excess Cu (2 mM) did find many repressed transcripts in the ergosterol pathway, including *UPC2* (Khemiri *et al.* 2020).

### Heme synthesis

We previously reported that growth of cells co-treated with fluconazole and Cu is rescued upon supplementation with heme (Hunsaker and Franz 2019a), a result that initially led to the hypothesis that Cu interferes with heme synthesis in fluconazole-treated cells. However, total cellular heme levels were two to three times higher in cells treated with fluconazole, regardless of Cu supplementation (Hunsaker and Franz 2019a).

**Figure 12.**
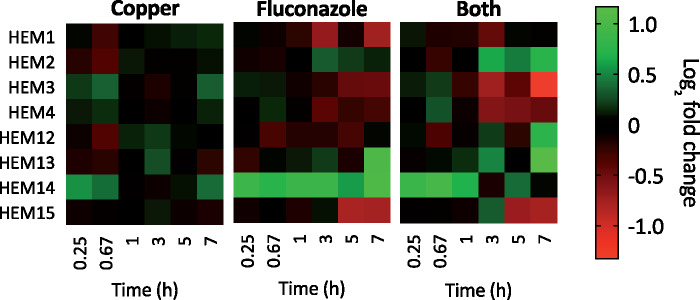
Heat maps showing log_2_ fold change expression for genes involved in heme biosynthesis, after 0.25, 0.67, 1, 3, 5, or 7 hours of treatment. Genes are listed in order as they appear in the pathway. Data are normalized to mRNA levels of untreated cells. Conditions: (Flu) = 50 µM, (CuSO_4_) = 10 µM in YPD medium.

*C. albicans* synthesizes heme through an 8-step pathway that begins with the formation of 5-aminolevulinc acid (ALA), catalyzed by 5-aminolevulinate synthase (gene product of *HEM1*), and ends with insertion of Fe into the porphyrin ring by ferrochelatase (*HEM15*) (Barupala *et al.* 2016). As shown in Figure 12, Cu alone had a modest but not particularly strong impact on the expression of genes in the heme biosynthetic pathway. In contrast, fluconazole had a more robust effect, with protoporphyrinogen oxidase (*HEM14*) standing out as the most upregulated gene of the series across all timepoints. Protoporphyrinogen oxidase catalyzes the penultimate step in the pathway, converting protoporphyrinogen IX to protoporphyrin IX. Interestingly, cells treated with Both induced *HEM14* within the first hour, but then expression declined, pointing to an effect of Cu swaying the inherent response to fluconazole. In addition to *HEM14*, Cu also had a fluconazole-reversing effect for *HEM1* and *HEM12*. In other steps, addition of Cu mostly matched or even accentuated the fluconazole response, especially at later time points, for example, induction of *HEM2* and *HEM13* and repression of *HEM3*, *HEM4*, and *HEM15*. These results intimate that fluconazole instigates disarray in the heme supply chain, which can be exacerbated by Cu.

## Conclusion

When *C. albicans* cells are pushed outside of homeostasis, either by elevated Cu levels or by fluconazole stress, they alter gene expression to neutralize the stress and restore homeostasis. This study was designed to uncover clues in the transcriptional response that might explain why slight elevation in Cu concentration diminishes *Candida’s* tolerance to fluconazole. Performing RNA-seq in a time course format allowed visualization of the dynamics of the cellular response, thereby removing limitations associated with analyzing only a single snapshot in time. We identified that fluconazole and Cu compete for control over gene expression during co-treatment with Both, and in the event that the transcriptomic response to Cu conflicts with the response to fluconazole, one of these treatments most often “wins out” to drive gene expression. In a significant number of cases, though, the transcriptomic response was uniquely driven by the combination treatment, underscoring the complexity and integrated nature of the cellular response.

Our analysis of differentially regulated genes has not revealed a “smoking gun” vulnerability created by the presence of Cu, but rather points to a wider array of “small cuts” that link *Candida’s* relative sensitivity to fluconazole with its ability to regulate Cu import. These small cuts showed up in the array of biological processes and pathways uniquely enriched by the combination treatment, as well as cases where the addition of Cu enhanced or countered the response to fluconazole on its own. Focusing specifically on metal homeostasis processes revealed that fluconazole itself induces changes in heme biosynthesis and metal homeostasis, especially for Fe and Cu, but also Zn and to a lesser extent Mn. The simultaneous induction of Cu import and storage with repression of Cu export in response to fluconazole suggests that *C. albicans* senses functional Cu deficiency and initiates processes to acquire and retain it. Our prior results showing that mutant strains lacking *MAC1*, *CTR1*, *CRP1*, *CUP1*, and *CCC2* are more susceptible to fluconazole than wild type are consistent with this idea that regulating Cu import helps *C. albicans* manage fluconazole stress (Hunsaker and Franz 2019a). Others have also reported increased susceptibility of a *MAC1* deletion strain to azole drugs, while also noting a reduced ability of this strain to adhere to and damage HT-29 enterocytes, which points to the importance of Cu regulation for virulence in a host (Khemiri *et al.* 2020).

It is important to emphasize that the low micromolar level of Cu used in our study did not impose an intrinsic stress to cells, but was sufficient to activate genes in the Cu regulon, thereby indicating that cells had to work to maintain Cu homeostasis. Responding to fluconazole stress could cause cells to become vulnerable to these otherwise sub-acute levels of Cu. Conversely, the effort of regaining Cu homeostasis may predispose cells to damage by fluconazole. As revealed by GO and KEGG enrichment analyses, fighting these dual fronts ultimately results in cells differentially regulating genes across a wide array of biological processes and pathways. Conditions of more severe Cu restriction or Cu overload would be likely to exacerbate these responses. Indeed, high levels of Cu have been shown to repress several genes in the ergosterol pathway which were largely unaffected under the low Cu used in our study (Khemiri *et al.* 2020).

Interestingly, regulation of genes associated with many of the same metabolic processes uncovered in our study are known to be essential for *Candida* to thrive in human hosts, and Cu homeostasis has been found as essential for this regulation. These results are important because the relative availability of metals to the pathogen varies based on the site and the progression of the infection. During *C. albicans* infections, Cu is limited in the kidney but available in excess in serum, an observation that may be a consequence of a global host response to infection and inflammation that signals reprioritization of Cu utilization (Besold *et al.* 2016; Culbertson *et al.* 2020). Thus, our finding that even small changes in Cu availability affect how the *C. albicans* transcriptome responds to fluconazole treatment could have implications for antifungal therapy to treat infections in which the niche Cu environment may be high or low.

## Data Availability

The authors state that all data necessary for confirming the conclusions presented in the article are represented fully within the article and accompanying Supplemental Material. Supplemental File S1 contains supplemental Materials and Methods describing the RNA isolation, preparation of libraries for sequencing, methods for processing RNA-seq data, methods for ICP-MS measurements, and Supplementary Figures S1, S2, and Supplementary Table S1. Full gene lists with log fold changes and *P*-values for all conditions and timepoints are provided in Supplemental File S2, tables of all the significant DEGs at each timepoint and their designated clusters are provided in Supplemental File S3, results from the GO and KEGG enrichment tests for all clusters at all timepoints are provided in Supplemental Files S4 and S5, respectively, definitions of GO Trim categories are in Supplemental File S6, and full GO Trim enrichment data is available in Supplemental File S7. The RNA sequencing data were deposited into NCBI’s Gene Expression Omnibus database under accession number GSE159545. Supplemental Material available at figshare: https://doi.org/10.25387/g3.14025080.
